# Atrial natriuretic peptide and three-dimensional echocardiography after transcatheter closure of atrial septal defect

**DOI:** 10.1186/1476-7120-6-35

**Published:** 2008-07-07

**Authors:** Jingdong Ding, Genshan Ma, Yaoyao Huang, Xiaoli Zhang, Biao Liu, Fengxiang Lu

**Affiliations:** 1 Department of Cardiology, Zhongda hospital & School of Clinical Medicine Southeast University, Nanjing, PR China; 2 Department of Nuclear Medicine, the First Affiliated Hospital of Nanjing Medical University, Nanjing, PR China; 3 Department of Cardiology, the First Affiliated Hospital of Nanjing Medical University, Nanjing, PR China

## Abstract

**Background:**

Atrial septal defect (ASD) accounts for 10% of all congenital heart lesions and represent the third most congenital cardiac defect seen in adults. Atrial natriuretic peptide (ANP) is an important regulator of the sodium and volume homeostasis. This study was designed to investigate the changes in plasma ANP concentrations and three-dimensional echocardiography (3DE) measurements of cardiac volume in patients with ASD during transcatheter closure of defect.

**Methods:**

Plasma ANP concentrations and transthoracic 3DE measurements of right ventricular volume were performed in 46 patients with ASD before closure, and at 3 days after closure. 22 healthy subjects matched for age, sex served as control subjects.

**Results:**

The 46 patients (20 men, 26 women; mean age 26.32 ± 13.28, range 6 to 63 years) were diagnosed to secundum ASD (the stretched diameters of ASD were from 9~36(25.34 ± 7.80 mm), and had been successfully placed Amplatzer septal occluder (the sizes of occluder were from 11 to 40 mm). The results showed that compared with control subjects, plasma ANP concentrations were elevated in patients with ASD. Plasma ANP concentrations positively correlated significantly with pulmonary artery pressure (PAP) (r = 0.74, *p *< 0.05) and 3DE measurements of cardiac volumes (right ventricular end-diastolic (r = 0.50, *p *< 0.05) and end-systolic volume (r = 0.50, *p *< 0.05) and negatively correlated with RVEF (r = -0.38, *p *< 0.05). Transthoracic 3DE measurements of right ventricular volume and plasma ANP concentrations decreased significantly at 3 days after closure (*p *< 0.05) compared with it before closure.

**Conclusion:**

Plasma ANP concentrations were markedly elevated in patients with pulmonary arterial hypertension and right ventricular volume overload and decreased significantly after closure of ASD. This study suggested that ANP may help to identify patients with ASD complicated by pulmonary arterial hypertension and right ventricular volume overload that demanded early intervention and may become effective marker for evaluating changes in cardiac load after transcatheter ASD closure.

## Background

Atrial septal defect (ASD) accounts for 10% of all congenital heart lesions and represent the third most congenital cardiac defect seen in adults[1]. This lesion is sometimes associated with the development of pulmonary arterial hypertension (PAH), right ventricular volume overload, congestive heart failure and atrial arrhythmias [1].

Surgical closure of the defect aims at relieving the heart and pulmonary circulation from the haemodynamic burden. Although patients benefit from surgical occlusion of the defect, they still have cardiovascular morbidity after the operation [1]. A new technique using the Amplatzer septal occluder was developed for the transcatheter closure of ASD [1-4]. This device is simple in construction, easy to deploy, and can be withdrawn and repositioned many times. It carries the advantages of fewer complications, avoidance of cardioplegia and cardiopulmonary bypass, shorter hospitalization, reduced need for blood products, and less patient discomfort. Although satisfactory results have been reported with transcatheter closure of ASD, there are some complications in this procedure. The type and rate of complications are different among different devices. Chessa et al [4] reported the embolization/malposition was the most common complication. Arrhythmias were the next most common complications. The other complications included right iliac vein dissection; groin hematoma, retropharynx hemorrhage.

ANP, a cardiac neurohormone secreted in the right atrium in response to increased right atrial pressure as well as volume loads [5,6]. It is an important regulator of the sodium and volume homeostasis[7]. It may be elevated in patients with congenital and acquired heart disease [8].

The present study was designed to evaluate the correlation between plasma ANP concentrations and PAP and right ventricular volume overload, and to investigate the changes in plasma ANP concentrations in patients with ASD during transcatheter closure of defects.

## Methods

### Study subjects

46 secundum ASD patients (20 men, 26 women; mean age 26.32 ± 13.28, range 6 to 63 years) and 22 healthy volunteers matched for sex and age (12 men, 10 women; mean age 24.81 ± 9.04, range 16 to 43 years) who served as control subjects were included in the study. We diagnosed all ASD patients by echocardiography. This study protocol was approved by the institutional ethics committee for human subjects. Informed consent was obtained for each study subject prior to participation in the study.

### Blood sampling and assay for ANP [9]

Blood samples were drawn from peripheral vein before and 3 days after transcatheter closure. Blood was immediately transferred into chilled glass tubes containing 2% disodium ethylenediamine tetraacetic acid (1 mg ml^-1^) and aprotinin (500 U ml^-1^) and centrifuged immediately at 3000 rpm for 10 min at 4°C. The collected plasma samples were then stored at -70°C until final analysis. Plasma ANP concentrations were measured directly with specific immunoradiometric assay kits (RIA ANP assay kit, Radioimmunoassay Center, General Hospital of Chinese PLA, Beijing, P.R. China).

### Three-dimensional echocardiographic image acquisition and volume calculation [10,11]

**Figure 1:**
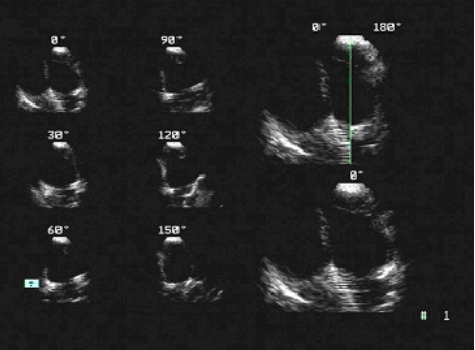
Graph of 3DE acquisition in a patient with ASD after transcatheter closure.

**Figure 2:**
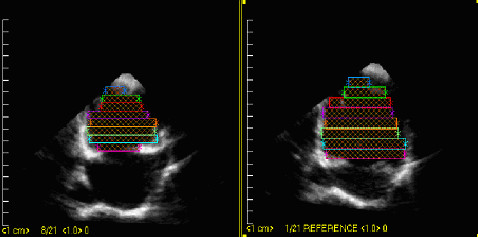
General views of 3DE reconstruction of the RV and measurement of RV volume and RVEF in a patient with ASD [Diastolic phase (left), Systolic phase (right)].

Transthoracic echocardiographic examination was undertaken with the patient lying supine or in the left lateral semi-recumbent position. Sedation was not used. Images were obtained with commercially available ultrasound equipment (SONOS 5500 and 7500, Hewlett-Packard Company, Andover, Mass) and saved in digital form on the hard disk of the ultrasound scanner. Standard parasternal, apical, and subcostal views were used. 3DE was done using rotational image acquisition from the apical view for right ventricle, with ECG and respiratory gating (Fig 1). 3DE images were reconstructed by Tom-Tec EchoView 4.2 (TomTec Imaging Systems GmHb, Munich, Germany). The right ventricular end-diastolic volume (RVEDV) and end-systolic volume (RVESV) were measured from each 3DE data set with "summation of disks" method, and stroke volume and ejection fraction (EF) was derived from those (Fig 2).

### Transcatheter closure of atrial septal defect [2]

**Figure 3:**
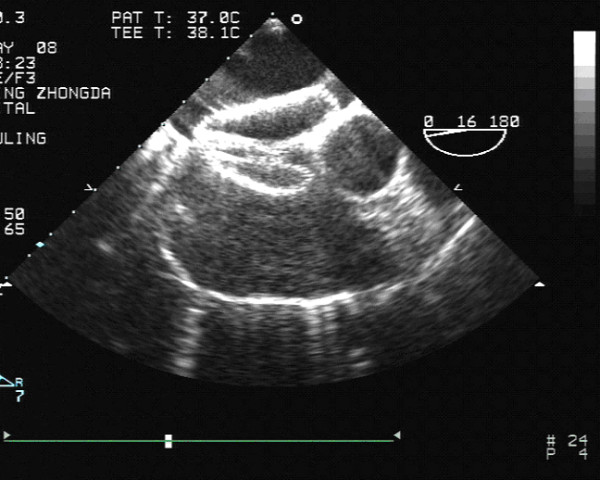
Transesophageal echocardiographic image of transcatheter ASD closure.

Briefly, implantation of the Amplatzer device was performed under general or local anesthesia with the guidance of transoesophageal or transthoracic echocardiography and fluoroscopy. First, a standard right heart catheterization was performed. Baseline hemodynamic variables including PAP, mean right atrial pressure and right ventricular end-diastolic pressure were measured in all patients. The stretched diameters of the ASD were measured by balloon sizing. The device for closing the ASD was selected to match the stretched diameter or 1~2 mm larger. After balloon sizing, the introducing sheath was inserted into the left atrium. Under fluoroscopic control, the delivery system, already loaded with the device, was introduced into the left atrium through the introducing sheath. After the waist and distal disc were deployed in the middle left atrium, the delivery system was gently pulled back against the atrial septum. Only when the waist was correctly positioned in the ASD, was the right atrial disc (proximal disc) deployed in the right atrium by slowly retracting the introducing sheath while keeping gentle traction on the cable. After the proper position was verified by transoesophageal or transthoracic echocardiography and fluoroscopy, the device was released (Fig 3). Aspirin treatment (3~5 mg/kg) was started 24 hours before transcatheter closure and continued for six months.

### Statistics

The results are expressed as mean ± standard deviation. Two-tailed, unpaired Student's *t *tests were used for comparison of variables between two groups. The multiple linear stepwise regression analysis was used to evaluate the associations between ANP and PAP, 3DE measurements. Treatment effects of transcatheter closure of defect were analyzed with the Student *t *test for paired samples. *P*-values < 0.05 were considered statistically significant. The statistical analyses were done using SPSS statistical software (SPSS for Windows, version 10.0, 1999, SPSS Inc, Chicago, IL).

## Results

Transcatheter closure of ASD and three-dimensional echocardiographic examinations were successfully performed in all patients, achieving a complete occlusion 3 days postoperatively in 100% of patients, and there were no complications, such as arrhythmia, embolization, or cardiac tamponade after the procedure. All patients had a single device implanted. The sizes of occluder were from 11 to 40 mm. The acquisition time of the echocardiographic data ranged from 8 to 10 minutes. Off-line image processing and analysis required on average 45 minutes.

### Clinical characteristics of patients with ASD and control subjects

The Clinical characteristics of patients with ASD and control subjects were listed in Table 1. There were no significant differences between the two groups with regard to age, sex.

**Table 1:** Clinical characteristics of study subjects

	Patients with ASD (n = 46)	Control subject (n = 22)	*p * Value
Age(years)	26.32 ± 13.28	24.81 ± 9.04	NS
Sex(M/F)	20/26	12/10	NS
ASD (TEE) (mm)	27.42 ± 5.92		
ASD (balloon) (mm)	25.34 ± 7.80		
PAP(mmHg)	36.48 ± 10.30		
RVEDV(ml)	106.54 ± 25.97	69.78 ± 10.46	<0.05
RVESV(ml)	59.73 ± 17.59	33.84 ± 7.18	<0.05
RVEF(%)	44.10 ± 4.72	51.92 ± 5.35	<0.05
ANP(ng/L)	156.42 ± 75.10	82.85 ± 17.80	<0.05

Data are presented mean value ± SD or number (%). ASD = atrial septal defect; TEE = transesophageal echocardiography; RVEDV = right ventricular end-diastolic volume; RVESV = right ventricular end-systolic volume; RVEF = right ventricular ejection fraction; ANP = atrial natriuretic peptide

### Plasma ANP concentrations and 3DE measurements

Plasma ANP concentrations and 3DE measurements of cardiac volume were shown in Table 1. Compared with it in control subjects, plasma ANP concentrations before closure were significantly elevated. The RVEDV and RVESV were enlarged in patients with ASD compared with those in control subjects, resulting in a marked decrease of the RVEF from control subjects.

### Correlation between Plasma ANP concentrations with PAP

**Figure 4:**
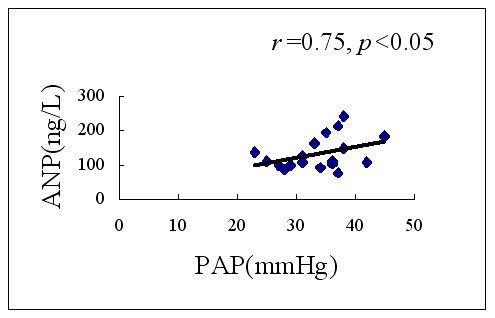
Correlation between plasma ANP concentrations with PAP.

PAP was 38.54 ± 11.02 mmHg among the 46 patients with ASD in whom this was measured during transcatheter closure of defects. There were significant positive correlation between plasma ANP concentrations and PAP (*r *= 0.74; *p *< 0.05) (Fig 4).

### Correlation between Plasma ANP concentrations and 3DE measurements

**Figure 5:**
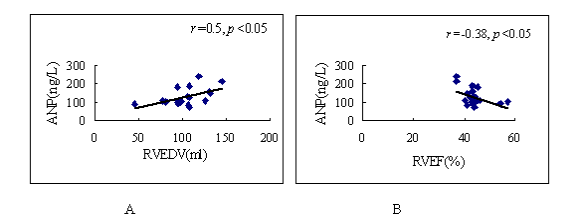
Correlation between plasma ANP concentrations with EVEDV (A), RVEF (B).

Before closure, in 46 patients with ASD, the plasma ANP concentrations positively correlated with RVEDV (*r *= 050; *p *< 0.05) and RVESV (*r *= 050; *p *< 0.05); and it negatively correlated with RVEF (*r *= -0.38; *p *< 0.05) (Fig 5).

### Effect of transcatheter closure of ASD on plasma ANP concentrations and 3DE measurements

**Figure 6:**
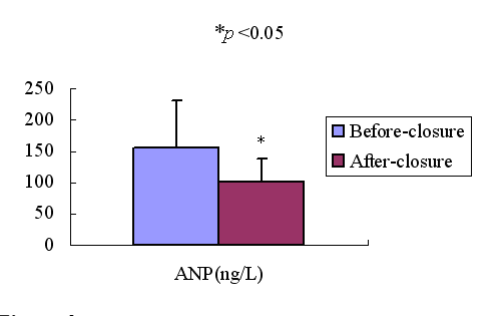
Bar graph showed plasma ANP concentrations in patients with ASD before and after closure. *p < 0.05.

**Figure 7:**
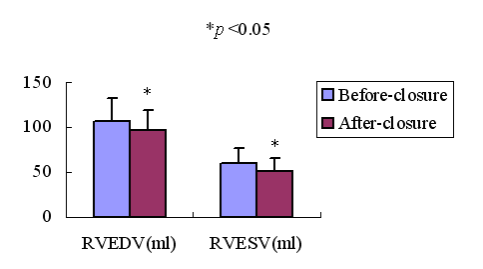
Bar graph showed RVEDV and RVESV in patients with ASD before and after closure. *p < 0.05.

**Figure 8:**
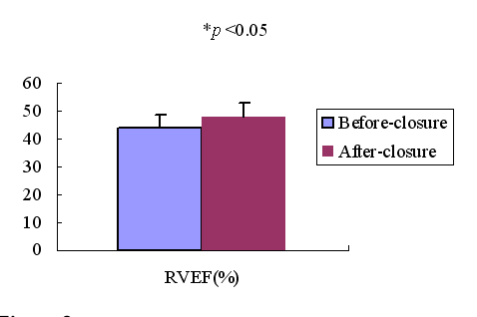
Bar graph showed RVEF in patients with ASD before and after closure. *p < 0.05.

46 patients with ASD underwent transcatheter closure of ASD, plasma ANP concentrations and 3DE measurements were measured before and 3 days after transcatheter closure of ASD with the Amplatzer septal occluder. Fig 6, Fig 7 and Fig 8 showed the changes of plasma ANP concentrations, right ventricular volume and ejection fraction after transcatheter closure of ASD. After closure, the plasma ANP concentrations decreased from 156.42 ± 75.10 ng/L to 101.78 ± 35.72 ng/L (*p *< 0.05), the RVEDV, RVESV decreased (*P *< 0.05), and the RVEF (*P *< 0.05) increased.

## Discussion

Previous studies evaluated the RV geometric change response to transcatheter closure of ASD by the measurement of a single RV dimension in a long- or short-axis view, or both [3,12,13]. RV dimension does not adequately reflect the complex geometry of the right ventricle, and there are no clear landmarks to ensure that this dimension is measured in the same anatomic location across serial studies. We used a three-dimensional echocardiographic volumetry to calculate RV volumes. This model might offer even more accurate assessment of right-sided chamber volumes. Thus, the measurement of RV volumes in this study allowed much more confidence than the measurement of a simple linear dimension.

In this study, we showed that (1) plasma ANP concentrations were elevated in patients with ASD regardless of the presence of PAH, (2) plasma ANP concentrations were correlated positively with PAP, RVEDV and RVESV; and correlated negatively with RVEF, and (3) after transcatheter closure of ASD, plasma ANP concentrations were markedly decreased, the RVEDV, RVESV decreased and the RVEF increased markedly.

These findings are consistent with previous reports on ANP that showed a positive correlation between plasma ANP concentrations and Qp/Qs and a positive correlation between plasma ANP and PAP in patients with different types of congenital heart disease, including ASD [5,14,15].

ANP is a 28 amino acid peptide and produced primarily in the cardiac atria [8], stimulated by atrial stretch, for example, volume overloading. ANP is a biologically active peptide with natriuretic and diuretic activities. In our study, plasma ANP concentrations were correlated positively with PAP and RVEDV in patients with ASD. These results imply that ANP may be released in response to right heart volume overload caused by the left-to right shunt in patients with ASD. Possible pathophysiologic significance of ANP increase in patients with ASD may relate to their pulmonary vasodilative effect. Increased ANP concentrations in patients with ASD correlated positively with PAP may play an important role in protecting the pulmonary bed.

In the last 50 years cardiac catheterization has changed its primary role from a diagnostic to that of a therapeutic procedure. Most recently, ASD transcatheter occlusion techniques have become an alternative to surgical procedure using cardiopulmonary bypass [4]. Our study showed that the plasma ANP concentrations decreased markedly at 3 days after transcatheter closure and there was a significant decrease in right ventricular volume by 3 days after transcatheter closure. Muta et al [16] demonstrated that the plasma ANP concentrations at 1 month after ASD closure decreased and were similar to control values. Schussler et al [17] demonstrated right ventricular volumes decreased by 22%, 30%, and 41% at 1 day, 1 month, and 6 months, respectively, after successful transcatheter closure of large ASDs. Right atrial areas decreased by 5%, 23%, and 26%, respectively, over the same time. Presumably, the early reduction in right heart size is secondary to removal of the left-to-right shunt and reduction of pro-load [3]. Right ventricular volumes continued to improve for up to 6 months, suggesting an ongoing remodeling process [17]. The mechanism of ANP decreases may reflect a reduction of volume overload of right heart.

Although it became readily apparent that 3DE provides more accurate and reliable measurements of right ventricular volume and function, the complex image acquisition and off-line data processing have limited the use of 3DE in daily clinical practice.

## Conclusion

The ASD was found to be associated with increased right ventricular volume, pulmonary arterial hypertension and was accompanied by increased plasma ANP concentrations, probably due to long-standing atrial stretch, when compared with controls. The change in plasma ANP concentrations may reflect changes in cardiac load during transcatheter closure of ASD. Thus plasma ANP concentrations can reflect pressure and volume loads to the pulmonary artery and right ventricle, and may be helpful in identifying patients with ASD and pulmonary arterial hypertension that demands early intervention.

## Authors' contributions

JDD, GSM and FXL participated to the design of the study. JDD and GSM were responsible for collection of data and drafting of manuscript. JDD performed the statistical analysis and revised the manuscript critically for important intellectual content. YYH and XLZ performed three-dimensional echocardiography, BL and JDD carried out immunoradiometric assay. All authors read and approved the final manuscript.
